# *N*′-(Cyclo­hexyl­carbon­yl)isonicotino­hydrazide

**DOI:** 10.1107/S1600536809027469

**Published:** 2009-07-18

**Authors:** H. S. Naveenkumar, Amirin Sadikun, Pazilah Ibrahim, Chin Sing Yeap, Hoong-Kun Fun

**Affiliations:** a School of Pharmaceutical Sciences, Universiti Sains Malaysia, 11800 USM, Penang, Malaysia; b X-ray Crystallography Unit, School of Physics, Universiti Sains Malaysia, 11800 USM, Penang, Malaysia

## Abstract

In the title compound, C_13_H_17_N_3_O_2_, the mean plane of the cyclo­hexane ring forms a dihedral angle of 33.12 (5)° with the pyridine ring. The two O atoms are twisted away from each other, as indicated by the C—N—N—C torsion angle of −74.97 (9)°. In the crystal structure, mol­ecules are linked into a three-dimensional network by inter­molecular N—H⋯N, N—H⋯O and C—H⋯O hydrogen bonds. The structure is also stabilized by C—H⋯π inter­actions.

## Related literature

For bond-length data, see: Allen *et al.* (1987 ▶). For applications of isoniazid derivatives, see: Janin (2007 ▶); Maccari *et al.* (2005 ▶); Slayden & Barry (2000 ▶). For the preparation, see: Besra *et al.* (1993 ▶). For the stability of the temperature controller used for the data collection, see: Cosier & Glazer (1986 ▶).
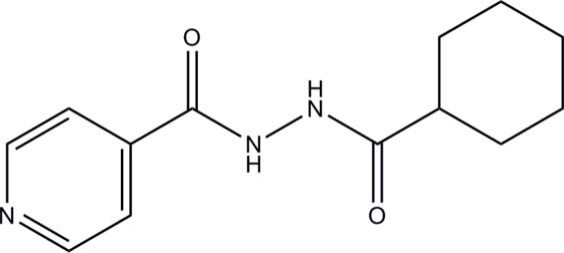


## Experimental

### 

#### Crystal data

C_13_H_17_N_3_O_2_
*M* *_r_* = 247.30
Orthorhombic, 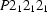

*a* = 9.1184 (2) Å
*b* = 11.5989 (2) Å
*c* = 12.1684 (2) Å
*V* = 1286.97 (4) Å^3^
*Z* = 4
Mo *K*α radiation
μ = 0.09 mm^−1^
*T* = 100 K
0.60 × 0.40 × 0.33 mm

#### Data collection

Bruker SMART APEXII CCD area-detector diffractometer
Absorption correction: multi-scan (**SADABS**; Bruker, 2005 ▶) *T*_min_ = 0.941, *T*_max_ = 0.971
27969 measured reflections
3210 independent reflections
3124 reflections with *I* > 2σ(*I*)
*R*_int_ = 0.022

#### Refinement

*R*[*F* ^2^ > 2σ(*F* ^2^)] = 0.032
*wR*(*F* ^2^) = 0.084
*S* = 1.11
3210 reflections
231 parameters
H atoms treated by a mixture of independent and constrained refinement
Δρ_max_ = 0.25 e Å^−3^
Δρ_min_ = −0.34 e Å^−3^

### 

Data collection: *APEX2* (Bruker, 2005 ▶); cell refinement: *SAINT* (Bruker, 2005 ▶); data reduction: *SAINT*; program(s) used to solve structure: *SHELXTL* (Sheldrick, 2008 ▶); program(s) used to refine structure: *SHELXTL*; molecular graphics: *SHELXTL*; software used to prepare material for publication: *SHELXTL* and *PLATON* (Spek, 2009 ▶).

## Supplementary Material

Crystal structure: contains datablocks global, I. DOI: 10.1107/S1600536809027469/kj2133sup1.cif

Structure factors: contains datablocks I. DOI: 10.1107/S1600536809027469/kj2133Isup2.hkl

Additional supplementary materials:  crystallographic information; 3D view; checkCIF report

## Figures and Tables

**Table 1:** Hydrogen-bond geometry (Å, °)

*D*—H⋯*A*	*D*—H	H⋯*A*	*D*⋯*A*	*D*—H⋯*A*
N2—H1*N*2⋯N1^i^	0.911 (18)	2.095 (18)	2.9456 (10)	155.0 (16)
N3—H1*N*3⋯O2^ii^	0.900 (16)	1.854 (16)	2.7486 (9)	172.4 (14)
C2—H2⋯O1^iii^	1.017 (18)	2.527 (18)	3.4971 (11)	159.4 (14)
C4—H4⋯O1^iv^	0.948 (15)	2.502 (15)	3.3062 (10)	142.6 (12)
C10—H10*B*⋯*Cg*1^v^	1.01 (2)	2.78 (3)	3.7299 (14)	157.1 (16)
C13—H13*A*⋯*Cg*1^vi^	0.993 (15)	2.958 (16)	3.7039 (10)	132.7 (12)

Symmetry codes: (i) 
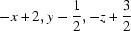
; (ii) 
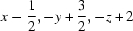
; (iii) 
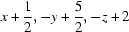
; (iv) 
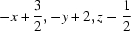
; (v) 
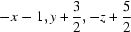
; (vi) 
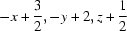
. *Cg*1 is the centroid of the C1/C2/N1/C3–C5 ring.

**Fig. 1.**
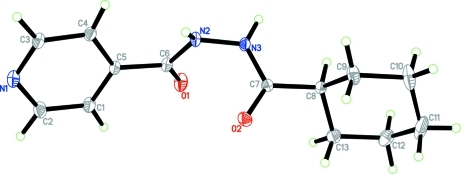
The molecular structure of the title compound with atom labels and 50% probability ellipsoids for non-H atoms.

## supplementary crystallographic information

### Comment

In the search of new compounds, isoniazid derivatives have been found to possess potential tuberculostatic activity (Janin, 2007; Maccari *et al.*, 2005; Slayden & Barry, 2000). As a part of a current work of synthesis of such derivatives, in this paper we present the crystal structure of the title compound which was synthesized in our lab.

Bond lengths and angles of the title compound (I), (Fig. 1) are within the normal range (Allen *et al.*, 1987). The mean plane of cyclohexane ring forms dihedral angle of 33.12 (5)° with the pyridine ring. The O1 and O2 atoms are twisted away from each other as is indicated by torsion angle C6–N2–N3–C7 [-74.97 (9)°]. In the crystal structure, the molecules are linked into three-dimensional network by the intermolecular N2—H1N2···N1, N3—H1N3···O2, C2—H2···O1 and C4—H4···O1 hydrogen bonds. The structure is also stabilized by C—H···π interactions (Table 1).

### Experimental

The isoniazid (INH) derivative was prepared following the procedure by literature (Besra *et al.*, 1993). Dry dichloromethane (30 ml) and 4-dimethylaminopyridine (4-DMAP) (1.2 eq) was added to cyclohexane carbonyl chloride followed by INH (1.1 eq). The reaction mixture was kept in an ice bath for 1 h and then left stirring under nitrogen overnight at room temperature. Dichloromethane (20 ml) was added to the reaction mixture, which was then washed with water, and the organic layer dried over anhydrous sodium sulphate. The solvent was removed under reduced pressure to afford the crude product which was purified by column chromatography and recrystallized from methanol to afford colorless crystals.

### Refinement

All hydrogen atoms were located from the difference Fourier map and refined freely. As there are not enough anomalous dispersion effects to determine the absolute configuration, 2499 Friedel pairs were merged before final refinement.

### Crystal data

**Table:**

C_13_H_17_N_3_O_2_	*F*(000) = 528
*M**_r_* = 247.30	*D*_x_ = 1.276 Mg m^−^^3^
Orthorhombic, *P*2_1_2_1_2_1_	Mo *K*α radiation, λ = 0.71073 Å
Hall symbol: P 2ac 2ab	Cell parameters from 9922 reflections
*a* = 9.1184 (2) Å	θ = 2.8–35.1°
*b* = 11.5989 (2) Å	µ = 0.09 mm^−^^1^
*c* = 12.1684 (2) Å	*T* = 100 K
*V* = 1286.97 (4) Å^3^	Block, colourless
*Z* = 4	0.60 × 0.40 × 0.33 mm

### Data collection

**Table:**

Bruker SMART APEXII CCD area-detector diffractometer	3210 independent reflections
Radiation source: fine-focus sealed tube	3124 reflections with *I* > 2σ(*I*)
graphite	*R*_int_ = 0.022
φ and ω scans	θ_max_ = 35.1°, θ_min_ = 2.4°
Absorption correction: multi-scan (*SADABS*; Bruker, 2005)	*h* = −14→14
*T*_min_ = 0.941, *T*_max_ = 0.971	*k* = −18→17
27969 measured reflections	*l* = −19→19

### Refinement

**Table:**

Refinement on *F*^2^	Primary atom site location: structure-invariant direct methods
Least-squares matrix: full	Secondary atom site location: difference Fourier map
*R*[*F*^2^ > 2σ(*F*^2^)] = 0.032	Hydrogen site location: inferred from neighbouring sites
*wR*(*F*^2^) = 0.084	H atoms treated by a mixture of independent and constrained refinement
*S* = 1.11	*w* = 1/[σ^2^(*F*_o_^2^) + (0.0593*P*)^2^ + 0.0754*P*] where *P* = (*F*_o_^2^ + 2*F*_c_^2^)/3
3210 reflections	(Δ/σ)_max_ < 0.001
231 parameters	Δρ_max_ = 0.25 e Å^−^^3^
0 restraints	Δρ_min_ = −0.34 e Å^−^^3^

### Special details

**Table:**

Experimental. The crystal was placed in the cold stream of an Oxford Cyrosystems Cobra open-flow nitrogen cryostat (Cosier & Glazer, 1986) operating at 100.0 (1)K.
Geometry. All esds (except the esd in the dihedral angle between two l.s. planes) are estimated using the full covariance matrix. The cell esds are taken into account individually in the estimation of esds in distances, angles and torsion angles; correlations between esds in cell parameters are only used when they are defined by crystal symmetry. An approximate (isotropic) treatment of cell esds is used for estimating esds involving l.s. planes.
Refinement. Refinement of F^2^ against ALL reflections. The weighted R-factor wR and goodness of fit S are based on F^2^, conventional R-factors R are based on F, with F set to zero for negative F^2^. The threshold expression of F^2^ > 2sigma(F^2^) is used only for calculating R-factors(gt) etc. and is not relevant to the choice of reflections for refinement. R-factors based on F^2^ are statistically about twice as large as those based on F, and R- factors based on ALL data will be even larger.

### Fractional atomic coordinates and isotropic or equivalent isotropic displacement parameters (Å^2^)

**Table:**

	*x*	*y*	*z*	*U*_iso_*/*U*_eq_	
O1	0.75737 (8)	1.01913 (6)	1.03928 (5)	0.02041 (13)	
O2	0.99005 (6)	0.79925 (5)	1.04401 (5)	0.01593 (11)	
N1	1.02861 (8)	1.26064 (6)	0.76325 (6)	0.01714 (12)	
N2	0.80178 (7)	0.89033 (6)	0.90156 (5)	0.01293 (11)	
N3	0.76418 (7)	0.79834 (6)	0.96907 (5)	0.01347 (11)	
C1	0.94236 (10)	1.18220 (7)	0.93422 (6)	0.01703 (14)	
C2	1.01495 (11)	1.26604 (7)	0.87318 (7)	0.01889 (14)	
C3	0.96940 (10)	1.16932 (7)	0.71250 (6)	0.01606 (13)	
C4	0.89624 (9)	1.08036 (7)	0.76639 (6)	0.01426 (12)	
C5	0.88303 (8)	1.08714 (6)	0.88042 (6)	0.01264 (12)	
C6	0.80847 (8)	0.99670 (6)	0.94875 (6)	0.01322 (12)	
C7	0.86549 (7)	0.75756 (6)	1.04018 (6)	0.01175 (11)	
C8	0.81852 (8)	0.65554 (6)	1.10913 (6)	0.01238 (11)	
C9	0.87614 (11)	0.54417 (7)	1.05682 (7)	0.02033 (15)	
C10	0.83192 (15)	0.43958 (8)	1.12611 (10)	0.0300 (2)	
C11	0.88525 (14)	0.45089 (10)	1.24449 (11)	0.0315 (2)	
C12	0.82967 (13)	0.56214 (10)	1.29578 (7)	0.02597 (18)	
C13	0.87382 (11)	0.66737 (8)	1.22748 (7)	0.02036 (15)	
H1	0.936 (2)	1.1925 (16)	1.0135 (14)	0.033 (4)*	
H2	1.062 (2)	1.3365 (15)	0.9082 (14)	0.032 (4)*	
H3	0.9786 (19)	1.1702 (14)	0.6350 (14)	0.027 (4)*	
H4	0.8560 (17)	1.0205 (13)	0.7227 (12)	0.018 (3)*	
H8	0.7101 (18)	0.6508 (14)	1.1062 (12)	0.021 (3)*	
H9A	0.837 (2)	0.5361 (17)	0.9849 (16)	0.042 (5)*	
H9B	0.9813 (19)	0.5498 (15)	1.0523 (14)	0.028 (4)*	
H10A	0.869 (2)	0.3690 (19)	1.0921 (16)	0.046 (5)*	
H10B	0.722 (3)	0.4297 (19)	1.1277 (16)	0.047 (5)*	
H11A	0.860 (2)	0.3808 (16)	1.2833 (14)	0.031 (4)*	
H11B	0.995 (3)	0.454 (2)	1.249 (2)	0.053 (6)*	
H12A	0.872 (2)	0.5711 (18)	1.3708 (15)	0.043 (5)*	
H12B	0.722 (2)	0.5602 (16)	1.2991 (13)	0.032 (4)*	
H13A	0.8388 (18)	0.7410 (13)	1.2598 (12)	0.018 (3)*	
H13B	0.985 (2)	0.6778 (15)	1.2258 (14)	0.030 (4)*	
H1N2	0.862 (2)	0.8724 (16)	0.8443 (15)	0.036 (5)*	
H1N3	0.6729 (18)	0.7705 (13)	0.9596 (12)	0.022 (3)*	

### Atomic displacement parameters (Å^2^)

**Table:**

	*U*^11^	*U*^22^	*U*^33^	*U*^12^	*U*^13^	*U*^23^
O1	0.0286 (3)	0.0174 (3)	0.0152 (2)	0.0016 (2)	0.0095 (2)	0.0005 (2)
O2	0.0107 (2)	0.0156 (2)	0.0215 (2)	−0.00104 (18)	−0.00008 (19)	0.0012 (2)
N1	0.0205 (3)	0.0136 (2)	0.0173 (3)	−0.0004 (2)	0.0028 (2)	0.0031 (2)
N2	0.0148 (2)	0.0104 (2)	0.0135 (2)	−0.00010 (19)	0.00287 (19)	0.00212 (19)
N3	0.0116 (2)	0.0128 (2)	0.0159 (2)	−0.00129 (19)	−0.0005 (2)	0.0046 (2)
C1	0.0258 (3)	0.0126 (3)	0.0127 (3)	−0.0015 (3)	0.0022 (2)	−0.0006 (2)
C2	0.0263 (4)	0.0129 (3)	0.0175 (3)	−0.0031 (3)	0.0013 (3)	0.0000 (2)
C3	0.0198 (3)	0.0155 (3)	0.0129 (3)	0.0002 (3)	0.0022 (2)	0.0030 (2)
C4	0.0179 (3)	0.0133 (3)	0.0115 (2)	−0.0005 (2)	0.0009 (2)	0.0011 (2)
C5	0.0158 (3)	0.0107 (3)	0.0115 (2)	0.0009 (2)	0.0018 (2)	0.0012 (2)
C6	0.0149 (3)	0.0116 (3)	0.0131 (3)	0.0016 (2)	0.0026 (2)	0.0014 (2)
C7	0.0111 (2)	0.0111 (2)	0.0131 (2)	0.0007 (2)	0.0008 (2)	−0.0001 (2)
C8	0.0125 (2)	0.0115 (3)	0.0131 (3)	0.0003 (2)	0.0005 (2)	0.0015 (2)
C9	0.0292 (4)	0.0117 (3)	0.0202 (3)	−0.0009 (3)	0.0069 (3)	−0.0015 (2)
C10	0.0457 (6)	0.0107 (3)	0.0336 (5)	−0.0014 (4)	0.0128 (4)	0.0013 (3)
C11	0.0324 (5)	0.0236 (4)	0.0386 (5)	0.0063 (4)	0.0018 (4)	0.0171 (4)
C12	0.0337 (5)	0.0278 (4)	0.0164 (3)	−0.0030 (4)	−0.0022 (3)	0.0080 (3)
C13	0.0275 (4)	0.0196 (3)	0.0139 (3)	−0.0032 (3)	−0.0030 (3)	0.0010 (2)

### Geometric parameters (Å, °)

**Table:**

O1—C6	1.2241 (9)	C7—C8	1.5125 (10)
O2—C7	1.2353 (9)	C8—C13	1.5321 (11)
N1—C3	1.3397 (11)	C8—C9	1.5329 (11)
N1—C2	1.3448 (11)	C8—H8	0.990 (16)
N2—C6	1.3622 (10)	C9—C10	1.5315 (13)
N2—N3	1.3895 (9)	C9—H9A	0.95 (2)
N2—H1N2	0.910 (19)	C9—H9B	0.963 (18)
N3—C7	1.3513 (9)	C10—C11	1.5260 (19)
N3—H1N3	0.900 (17)	C10—H10A	0.98 (2)
C1—C2	1.3912 (11)	C10—H10B	1.01 (2)
C1—C5	1.3917 (11)	C11—C12	1.5203 (18)
C1—H1	0.973 (18)	C11—H11A	0.967 (19)
C2—H2	1.017 (18)	C11—H11B	1.00 (3)
C3—C4	1.3927 (11)	C12—C13	1.5306 (13)
C3—H3	0.946 (17)	C12—H12A	0.997 (19)
C4—C5	1.3950 (10)	C12—H12B	0.98 (2)
C4—H4	0.948 (15)	C13—H13A	0.993 (15)
C5—C6	1.5014 (10)	C13—H13B	1.02 (2)
			
C3—N1—C2	117.26 (7)	C13—C8—H8	111.5 (8)
C6—N2—N3	117.22 (6)	C9—C8—H8	106.3 (9)
C6—N2—H1N2	120.2 (12)	C10—C9—C8	110.40 (7)
N3—N2—H1N2	115.2 (11)	C10—C9—H9A	109.4 (13)
C7—N3—N2	118.60 (6)	C8—C9—H9A	109.8 (13)
C7—N3—H1N3	126.1 (10)	C10—C9—H9B	110.4 (10)
N2—N3—H1N3	115.3 (10)	C8—C9—H9B	107.9 (11)
C2—C1—C5	119.18 (7)	H9A—C9—H9B	109.0 (17)
C2—C1—H1	118.0 (11)	C11—C10—C9	111.57 (9)
C5—C1—H1	122.8 (11)	C11—C10—H10A	111.1 (12)
N1—C2—C1	122.85 (8)	C9—C10—H10A	109.8 (12)
N1—C2—H2	114.5 (10)	C11—C10—H10B	107.9 (11)
C1—C2—H2	122.6 (10)	C9—C10—H10B	111.2 (12)
N1—C3—C4	124.18 (7)	H10A—C10—H10B	105.1 (17)
N1—C3—H3	114.5 (10)	C12—C11—C10	110.75 (8)
C4—C3—H3	121.3 (10)	C12—C11—H11A	115.7 (11)
C3—C4—C5	117.90 (7)	C10—C11—H11A	108.2 (11)
C3—C4—H4	117.7 (9)	C12—C11—H11B	106.4 (14)
C5—C4—H4	124.4 (9)	C10—C11—H11B	112.2 (15)
C1—C5—C4	118.62 (7)	H11A—C11—H11B	103.5 (18)
C1—C5—C6	117.98 (6)	C11—C12—C13	111.48 (8)
C4—C5—C6	123.41 (7)	C11—C12—H12A	109.6 (12)
O1—C6—N2	123.70 (7)	C13—C12—H12A	108.2 (12)
O1—C6—C5	121.48 (7)	C11—C12—H12B	109.3 (11)
N2—C6—C5	114.82 (6)	C13—C12—H12B	107.7 (11)
O2—C7—N3	121.04 (7)	H12A—C12—H12B	110.6 (16)
O2—C7—C8	123.07 (7)	C12—C13—C8	110.63 (7)
N3—C7—C8	115.81 (6)	C12—C13—H13A	112.7 (9)
C7—C8—C13	110.98 (6)	C8—C13—H13A	110.1 (9)
C7—C8—C9	109.38 (6)	C12—C13—H13B	111.5 (10)
C13—C8—C9	110.67 (7)	C8—C13—H13B	108.6 (9)
C7—C8—H8	107.9 (9)	H13A—C13—H13B	103.0 (14)
			
C6—N2—N3—C7	−74.97 (9)	N2—N3—C7—O2	−1.43 (11)
C3—N1—C2—C1	−0.37 (13)	N2—N3—C7—C8	−178.54 (6)
C5—C1—C2—N1	1.17 (14)	O2—C7—C8—C13	43.39 (10)
C2—N1—C3—C4	−0.43 (13)	N3—C7—C8—C13	−139.56 (7)
N1—C3—C4—C5	0.40 (13)	O2—C7—C8—C9	−79.00 (9)
C2—C1—C5—C4	−1.15 (12)	N3—C7—C8—C9	98.04 (8)
C2—C1—C5—C6	178.57 (8)	C7—C8—C9—C10	179.16 (8)
C3—C4—C5—C1	0.42 (12)	C13—C8—C9—C10	56.58 (10)
C3—C4—C5—C6	−179.29 (7)	C8—C9—C10—C11	−56.32 (12)
N3—N2—C6—O1	−14.45 (11)	C9—C10—C11—C12	55.80 (13)
N3—N2—C6—C5	166.16 (6)	C10—C11—C12—C13	−55.73 (12)
C1—C5—C6—O1	23.87 (12)	C11—C12—C13—C8	56.45 (11)
C4—C5—C6—O1	−156.42 (8)	C7—C8—C13—C12	−178.34 (7)
C1—C5—C6—N2	−156.72 (7)	C9—C8—C13—C12	−56.70 (10)
C4—C5—C6—N2	22.99 (11)		

### Hydrogen-bond geometry (Å, °)

**Table:**

*D*—H···*A*	*D*—H	H···*A*	*D*···*A*	*D*—H···*A*
N2—H1N2···N1^i^	0.911 (18)	2.095 (18)	2.9456 (10)	155.0 (16)
N3—H1N3···O2^ii^	0.900 (16)	1.854 (16)	2.7486 (9)	172.4 (14)
C2—H2···O1^iii^	1.017 (18)	2.527 (18)	3.4971 (11)	159.4 (14)
C4—H4···O1^iv^	0.948 (15)	2.502 (15)	3.3062 (10)	142.6 (12)
C10—H10B···Cg1^v^	1.01 (2)	2.78 (3)	3.7299 (14)	157.1 (16)
C13—H13A···Cg1^vi^	0.993 (15)	2.958 (16)	3.7039 (10)	132.7 (12)

Symmetry codes: (i) −*x*+2, *y*−1/2, −*z*+3/2; (ii) *x*−1/2, −*y*+3/2, −*z*+2; (iii) *x*+1/2, −*y*+5/2, −*z*+2; (iv) −*x*+3/2, −*y*+2, *z*−1/2; (v) −*x*−1, *y*+3/2, −*z*+5/2; (vi) −*x*+3/2, −*y*+2, *z*+1/2.
